# UniMR: A Plug‐and‐Play Framework of Automated Molecular Recognition for Scanning Tunneling Microscopy

**DOI:** 10.1002/advs.202516428

**Published:** 2025-12-15

**Authors:** Ziqiang Cao, Lingyin Zhang, Bingzheng Wu, Haonan Chen, Junhao Sun, Linghao Yan, Wenfei Li, Yangyang Wu, Zhifang Wang, Qigang Zhong, Lifeng Chi

**Affiliations:** ^1^ School of Computer Science and Technology Soochow University Suzhou 215123 China; ^2^ Research Center for Chemical Theory Department of Chemistry Fudan University Shanghai 200438 China; ^3^ State Key Laboratory of Bioinspired Interfacial Materials Science Institute of Functional Nano & Soft Materials (FUNSOM) Soochow University Suzhou 215123 China

**Keywords:** computer vision, molecular recognition, scanning tunneling microscopy

## Abstract

Scanning Tunneling Microscopy (STM) has become an indispensable tool for molecular surface chemistry, where recognizing molecules in STM images is a fundamental task. Human annotation requires a great deal of domain knowledge and is laborious, while current automated methods usually require training and focus on particular molecular systems. In this work, a training‐free universal molecular recognizer (UniMR) is developed that is widely applicable to various molecular systems and is tolerant of low‐resolution images. UniMR integrates off‐the‐shelf computer vision tools with a feature adaptive selection module. First, input images are normalized to ensure consistent molecular brightness and orientation. To address the varying complexity of molecular differentiation across different systems, an adaptive feature selection module is introduced. Specifically, each molecular image is represented by a pixel matrix for shallow structural features and CLIP embeddings for deep semantic features. A Gaussian Mixture Model (GMM) is followed to dynamically select the optimal representation. Finally, cosine similarity identifies molecules of the same type. UniMR is evaluated on five molecular systems with diverse imaging resolutions. The results demonstrate a significant improvement over previous methods, achieving an average F1‐score >0.9. This framework can serve as a versatile auxiliary tool for STM, advancing microscopic surface chemistry research.

## Graphical abstract

UniMR, a training‐free framework for automated molecular recognition in STM images. By integrating adaptive feature selection with CLIP embeddings and Gaussian Mixture Modeling, UniMR achieves robust performance across diverse molecular systems and low‐resolution conditions. Extensive validation demonstrates superior F1‐scores (0.93) over existing methods, highlighting its potential as a versatile tool for advancing surface science research.

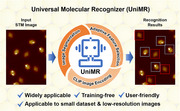

## 1 Introduction

Scanning Probe Microscopy (SPM) is an advanced microscopic imaging technique that employs an atomically sharp tip to traverse the sample surfaces, revealing the microscopic topography of the surfaces and detecting local chemical/physical properties.^[^ ^1^, ^2^, ^3^, ^4^, ^5^, ^6^, ^7^, ^8^, ^9^ ^]^ One of the most significant Scanning Probe Microscopes is Scanning Tunneling Microscope (STM).^[^ ^10^, ^11^, ^12^ ^]^ STM has been extensively used to characterize the structural and electronic properties of nanomaterials and molecules, offering an unparalleled level of spatial resolution.^[^ ^13^, ^14^, ^15^, ^16^, ^17^, ^18^, ^19^, ^20^, ^21^, ^22^ ^]^ In the realm of STM image analysis, the detection of target molecules is essential and fundamental. This task remains heavily dependent on the proficiency of seasoned experts, which is time‐consuming and error‐prone.^[^ ^23^, ^24^, ^25^, ^26^, ^27^, ^28^, ^29^ ^]^ Therefore, it hinges on the need for a highly efficient analytic tool capable of accurate and autonomous molecular recognition of STM images.

With the rapid development of the Computer Vision (CV) field, recent years have witnessed huge progress in deep learning based molecular recognition models. For example, Li et al.^[^ ^30^ ^]^ combined a designed data selection method and data augmentation techniques to train a Faster R‐CNN^[^ ^31^ ^]^ classifier, while Zhu et al.^[^ ^32^ ^]^ used Mask R‐CNN^[^ ^33^, ^34^ ^]^ to address the challenge of molecule detection, classification, and instance segmentation in bi‐component molecular nanostructures. Despite high precision, most current methods must be trained on a labeled dataset of molecules to be recognized in advance, hard to generalize for other molecules or STM images of different resolution. In addition, the mainstream works apply the convolutional neural networks (CNNs)^[^ ^35^ ^]^ as the backbone of the recognition model, while the Vision Transformer (ViT)^[^ ^36^ ^]^ has achieved state‐of‐the‐art performance on various CV tasks, including image classification,^[^ ^37^ ^]^ image‐text retrieval^[^ ^38^, ^39^ ^]^ and image‐to‐text generation.^[^ ^40^, ^41^, ^42^, ^43^ ^]^

**Figure 1:**
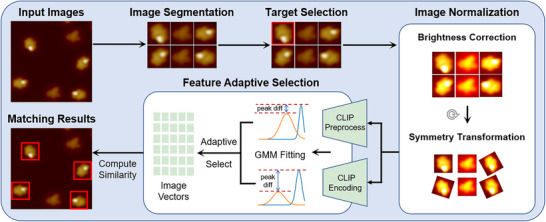
UniMR Workflow. Input: Users upload STM images and select target molecules. Normalization: The system normalizes all molecular images using dual image processors. Feature Extraction: A feature‐adaptive selection module converts the molecules into vector representations. Matching: Molecules with high vector similarity to the target are returned as identification results.

This paper proposes a Universal Molecular Recognizer (UniMR) to recognize molecules in any STM images at various resolutions. Compared with previous work in this area, our framework is purely unsupervised, meaning it can apply to any STM image without fine‐tuning. As shown in **Figure** **1**, our framework consists of a normalization module, a ViT image encoder and a feature adaptive selection module. Since molecular images are rotationally invariant and often exhibit significant variations in brightness due to different imaging conditions, we developed a normalization module comprising brightness correction and symmetry transformation to standardize and preprocess molecular images. Then, since some molecular systems are readily discernable from the images while others are not, we design a feature adaptive selection module. Specifically, we employ a state‐of‐the‐art ViT encoder CLIP^[^ ^44^ ^]^ to transform each molecular image into a semantic‐level encoded vector. Concurrently, we utilize the raw pixel matrix provided by CLIP preprocessing as shallow features. Then, using Gaussian Mixture Modeling (GMM), we fit the two feature sets to separate Gaussian distributions and dynamically switch between encoding methods based on which yields stronger peak separation. Finally, two molecules are recognized as the same if the cosine similarity of their feature vectors exceeds a threshold.

We test UniMR on five distinct molecular systems (molecule/substrate combinations: tert‐butyl substituted dibenzoperioctacene (tBu‐DBPO)/Au(111), dibenzoperihexacene (DBPH)/Cu(110), DBPH/Cu(111), DBPH/Au(111), 1‐bromo‐8‐phenylnaphthalene (BPN)/Cu(111)) across varying resolutions. Although some molecules exhibit only subtle STM contrast that requires discernment, even for human experts, our framework achieves robust automated identification with high efficiency and accuracy. It achieves an average F1 score of 0.93, significantly outperforming the baseline models (0.72). The performance advantage is statistically validated by a Wilcoxon signed‐rank test(p=0.00095). In addition, we systematically compared different encoder architectures and pre‐training strategies, observing that the current UniMR configuration with ViT encoder and CLIP pre‐training consistently surpasses alternatives such as ResNet‐50^[^ ^44^ ^]^ and Masked Autoencoder‐based models.^[^ ^45^ ^]^ Furthermore, as image resolution degrades, competing methods show substantial performance drops, whereas our model maintains consistent accuracy.

UniMR is an open‐source, lightweight tool designed for non‐expert users. As illustrated in Figure 1, the workflow requires just two simple steps: uploading a set of STM images and selecting target molecules. With no need for fine‐tuning, our framework offers a plug‐and‐play solution that can serve as a versatile auxiliary module for molecular recognition in STM systems.

## 2 Results and Discussion

### 2.1 Molecular Systems

**Figure 2:**
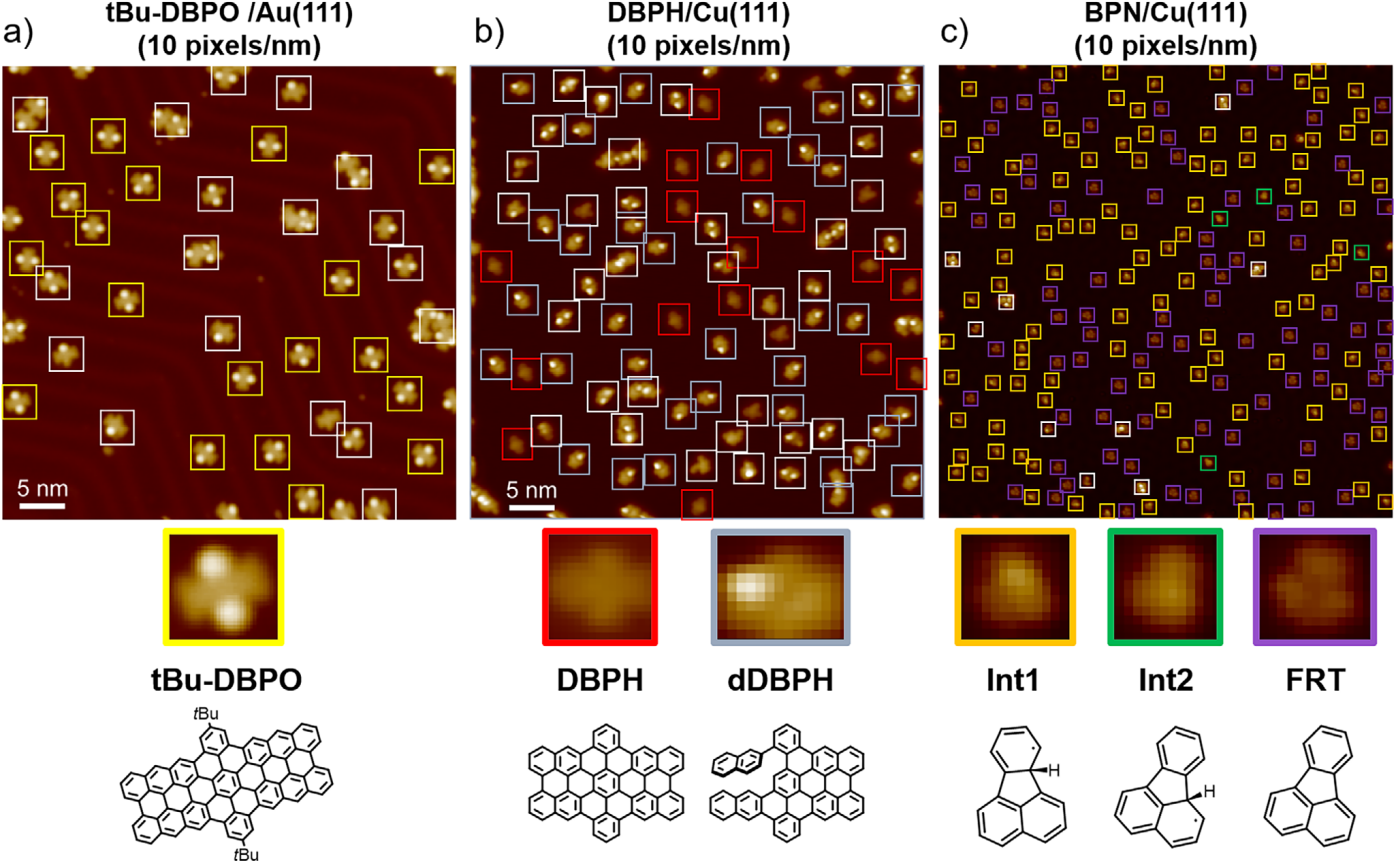
Representative STM images (10 pixels per nm resolution) of three different molecular systems: a) tBu‐DBPO on Au(111); b) DBPH on Cu(111); c) BPN on Cu(111). In the overview STM images (top row), colored square boxes mark the manually identified target molecules, while white boxes mark other non‐target molecules. The corresponding cropped STM images and structural formulas of the target molecules are shown in the lower panel.

Three different molecular systems were used to test the capability of our molecular recognition framework. The representative overview of STM images of the three molecular systems, together with the cropped single‐molecule STM images and chemical structures of the target molecules are shown in **Figure** **2**. Note that across all experiments in this work, molecular counts explicitly exclude edge molecules, treat overlapping molecular clusters as a single entity, and disregard imaging impurities. The first two molecular systems are nanographene synthesis on different surfaces, i.e., DBPH and tBu‐DBPO. DBPH was synthesized and characterized on three different metal surfaces, i.e., Cu(110), Cu(111) and Au(111). The apparent features of DBPH on the three surfaces show slight discrepancies because of the varied extent of molecule‐surface coupling. Our molecular recognition framework will automatically detect all product molecules in the STM image overview for the two molecular systems.

The STM images of DBPH and tBu‐DBPO were adapted from our previous work.^[^ ^19^ ^]^ The third molecular system, denoted with BPN/Cu(111) represents the complex multi‐step on‐surface reactions of BPN on a Cu(111) surface, where multiple reaction species (including intermediates Int1, Int2 and others, as well as the product fluoranthene (FRT)) coexist in an overview STM image of the sample surface. The high similarity of these molecular species makes it tedious to visually discriminate them one by one for statistical analysis, which would take a few days even for an experienced STM researcher. The STM images of BPN/Cu(111) were orginally acquired with a low‐temperature scanning probe microscope in the Group of Prof. Andre Schirmeisen at Justus Liebig University Giessen, Germany. Please check the previous paper for STM experimental details.^[^ ^29^ ^]^ The three similar molecular species, namely Int1, Int2 and FRT, can be distinguished by the presence/absence and intramolecular position of the protruding hydrogen atom, as corroborated by STM simulations^[^ ^46^, ^47^, ^48^, ^49^ ^]^ (see Figure S1, Supporting Information). Int1 and Int2 possess a protruding hydrogen atom, which appears as a brighter spot relative to the molecular backbone in STM images, while FRT is flat without protrusion. The difference between Int1 and Int2 lies in the atomic position of the protruding hydrogen atom, i.e., the hydrogen atom is attached to the phenyl group in Int1 but shifted to the naphthyl moiety in Int2. However, the ultra‐high similarity between Int1 and Int2 makes their distinction even more challenging.

Key characteristics of these molecular systems are summarized in **Table** **1**. Among them, the BPN/Cu(111) system presents the greatest complexity, containing three distinct target molecule types. Since high‐resolution imaging (e.g., 20 pixels per nm) remains cost‐prohibitive, most overview STM images are acquired at lower resolutions, making molecular recognition significantly challenging. Despite its simplicity, our image segmentation module achieves >97% detection accuracy for actual target molecules. The remaining failures are mainly attributed to overlapping instances where aggregated structures exceed the predefined size range for individual molecules.

**Table 1:** Statistics of image segmentation for different molecular systems. “Actual#” stands for the manually counted number of target molecules in each image, while “Inspection#” represents the number of molecules detected by the image segmentation module. Others represent non‐target molecules.

	Molecule	Inspection#	Actual#
tBu‐DBPO/Au(111) (10 pixels per nm)	tBu‐DBPO	19	19
	Others	10	18
DBPH/Au(111) (10 pixels per nm)	DBPH	42	42
	Others	8	21
DBPH/Cu(110) (25 pixels per nm)	DBPH	8	8
	dDBPH	4	4
	Others	15	15
DBPH/Cu(111) (10 pixels per nm)	DBPH	15	16
	dDBPH	31	34
	Others	30	34
BPN/Cu(111) (10 pixels per nm)	Int1	293	303
	Int2	21	21
	FRT	224	231
	Others	14	28
BPN/Cu(111) (20 pixels per nm)	Int1	164	169
	Int2	2	2
	FRT	28	28
	Others	14	17

### 2.2 Overall Recognition Pipeline

As shown in Figure 1, the overall molecular recognition pipeline consists of the following steps.

*Image Segmentation*: Since standard unsupervised methods like watershed^[^ ^50^ ^]^ showed limited effectiveness in reliably identifying overlapping molecules, we adopted a contour‐based detection approach. Specifically, we simply set the image patches with a brightness greater than a certain threshold as molecules, and implement this feature through the “findContours function” in OpenCV^[^ ^51^ ^]^ (Figure S2, Supporting Information).

*Target Molecule Selection*: Users choose one or more kinds of target molecules that need to be identified from the overview STM images.

2.2.1

*Normalization*: The normalization module is a critical preprocessing stage in our pipeline, designed to standardize molecular images for enhanced matching accuracy and consistency. It consists of two key components:

- Brightness Correction (BC) normalizes image luminance to a target value of 120 (±20).
- Symmetry Transformation (ST) aligns molecular postures through 36 rotational increments (10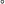 per step) and mirroring operations.

*Feature Adaptive Selection*: The feature adaptive selection module dynamically determines whether to employ CLIP embeddings through rigorous statistical evaluation of encoded data, optimizing representations based on molecular distinguishability. For highly distinguishable systems, it utilizes pixel‐level shallow representations, while adopting semantic‐level deep representations for less distinguishable cases. This selection process employs GMM‐based analysis that fits encoded data into two Gaussian distributions, with the peak difference between distributions serving as the core comparison metric. The encoding method corresponding to the distribution with greater peak difference is selected as it provides more discriminative information. By adaptively choosing the most informative embedding method, the module ensures optimal representation for subsequent processing across diverse molecular systems.

*Molecular Matching*: After determining the optimal encoding method through the feature adaptive selection module, we encode both the target molecule and the query molecule into their respective vector representations. Using cosine similarity as our matching metric, we classified any molecule exceeding a predefined similarity threshold as belonging to the target molecule category.

Because all modules are derived from ready‐made models, our solution does not require training or manual data annotation, achieving plug‐and‐play functionality on any molecular image set. This approach uniquely supports cross‐experimental molecular identification, as demonstrated later in our DBPH case study, significantly enhancing the analysis and generalization of similar chemical reaction patterns.

### 2.3 Performance of UniMR

We conducted molecular recognition experiments on five different molecular systems shown in Table 1 to verify the effectiveness of the proposed methods. Since we focus on the plug‐and‐play scenario, the common supervised CNN approaches^[^ ^30^, ^32^ ^]^ are not applicable. Instead, we introduce the following general baselines for comparison.

*Zernike*: Hellerstedt et al.^[^ ^52^ ^]^ uses the Zernike polynomial basis set^[^ ^53^ ^]^ to provide a “fingerprint” of coefficients representing every molecule. Zernike polynomials are robust in response to molecular rotations.

*SAM*: Kirillov et al.^[^ ^45^ ^]^ proposes the Segment Anything Model (SAM), which provides a pipeline for any object segmentation and clustering. However, our preliminary experiments found that SAM tends to chop molecules. Therefore, we just used SAM equipped with the ViT‐H/14 backbone as a molecular image encoding strategy for comparison with UniMR.

**Figure 3:**
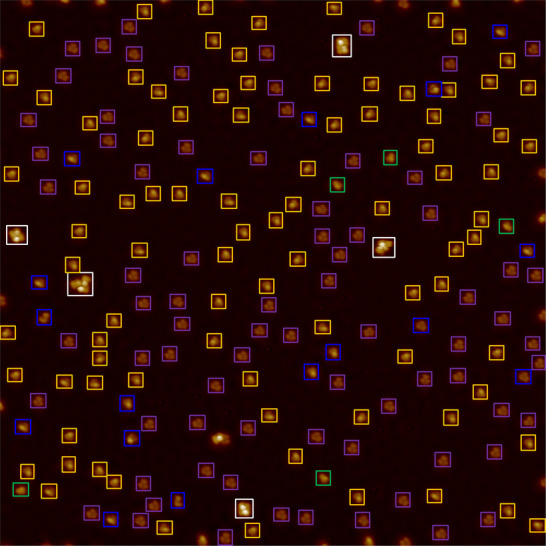
The UniMR molecular recognition results for a low‐resolution (10 pixels per nm) overview STM image of BPN/Cu(111). The blue square boxes represent incorrect matches, while the other colors represent correct matches (Yellow: Int1; Green: Int2; Purple: FRT; White: Others). Precision, recall, and F1‐score are 0.96, 0.93, and 0.94, respectively. The errors are mainly derived from the high similarity of molecules Int1 and Int2.

The molecular recognition performance of different methods is shown in **Tables** 2, 3, 4, 5. As can be seen, UniMR outperforms all the other approaches to a large extent, regardless of the type of molecular systems or the level of image resolution. A sample of the UniMR molecular recognition results is shown in **Figure** **3**, and the rest of the results can be found in the (Figures S4 and S5, Supporting Information). Apart from only one type of molecules (Int2), the average recognition F1‐score of our model in all experiments reaches 0.95, while Zernike is 0.74. For DBPH on multiple substrates (Cu(110), Cu(111), Au(111)), UniMR's average F1‐score reached 0.93 (vs Zernike's 0.75), with precision consistently above 0.84 and recall near 1.00 across all cases. Notably, the precision on Au(111) was marginally lower (0.84), likely due to interference from its characteristic herringbone reconstruction, as shown in Figure S3f (Supporting Information), which can introduce local imaging artifacts. Despite this minor variation, the results clearly demonstrate that the substrate type has no direct or substantial restriction on the recognition capability of UniMR. Comparing the molecular recognition results for BPN/Cu(111) between high (20 pixels per nm) and low (10 pixels per nm) imaging resolutions (see **Table** **4** vs Table 5), we observe that our method maintains relatively stable performance (0.95→0.91). Finally, the comparison between UniMR and SAM+ clearly demonstrates that CLIP outperforms SAM in molecular image encoding.

**Table 2:** Molecular recognition performance on tBu‐DBPO/Au(111).

		Precision	Recall	F1‐score
Zernike	tBu‐DBPO	0.58	1.00	0.73
UniMR	tBu‐DBPO	**0.86**	1.00	**0.93**

**Table 3:** Molecular recognition performance on DBPH, representing the overall statistics for dDBPH on Cu(111) and DBPH on Cu(110), Cu(111) and Au(111).

			Precision	Recall	F1‐score
Zernike	dDBPH	Cu(111)	0.51	1.00	0.67
		Cu(110)	0.33	0.33	0.33
	DBPH	Cu(110)	0.47	1.00	0.64
		Cu(111)	0.55	0.79	0.65
		Au(111)	0.75	1.00	0.86
	Average		0.61	0.95	0.74
UniMR	dDBPH	Cu(111)	0.91	0.97	0.94
		Cu(110)	1.00	1.00	1.00
	DBPH	Cu(110)	0.89	1.00	0.94
		Cu(111)	0.88	1.00	0.93
		Au(111)	0.84	1.00	0.91
	Average		**0.88**	**0.99**	**0.93**

**Table 4:** Molecular recognition performance on BPN/Cu(111) with high imaging resolution (20 pixels per nm). SAM+ indicates the SAM encoding after our normalization modules.

		Precision	Recall	F1‐score
Zernike	Int1	0.98	0.57	0.72
	Int2	0.05	1.00	0.10
	FRT	1.00	0.75	0.86
	Average	0.97	0.60	0.73
SAM	Int1	0.91	0.24	0.38
	Int2	0.05	0.50	0.08
	FRT	0.42	0.18	0.25
	Average	0.83	0.23	0.36
SAM+	Int1	0.84	0.26	0.39
	Int2	0.04	0.50	0.07
	FRT	0.31	0.18	0.23
	Average	0.76	0.25	0.37
UniMR	Int1	0.99	0.92	0.96
	Int2	0.07	0.50	0.12
	FRT	1.00	1.00	1.00
	Average	**0.98**	**0.93**	**0.95**

**Table 5:** Molecular recognition performance on BPN/Cu(111) with low imaging resolution (10 pixels per nm).

		Precision	Recall	F1‐score
Zernike	Int1	0.87	0.68	0.76
	Int2	0.12	0.62	0.19
	FRT	0.96	0.45	0.61
	Average	0.88	0.58	0.68
SAM	Int1	0.54	0.33	0.41
	Int2	0.03	0.14	0.06
	FRT	1.00	0.08	0.15
	Average	0.71	0.22	0.29
SAM+	Int1	0.68	0.33	0.44
	Int2	0.04	0.14	0.06
	FRT	1.00	0.27	0.42
	Average	0.79	0.30	0.42
UniMR	Int1	0.94	0.90	0.92
	Int2	0.19	0.57	0.29
	FRT	1.00	0.90	0.95
	Average	**0.93**	**0.89**	**0.91**

*Statistical Analysis*: Performance metrics (precision, recall, F1‐score) represent single measurements without standard deviations. Sample sizes correspond to Table 1 “inspection” counts. We evaluated UniMR against the Zernike baseline using Wilcoxon signed‐rank tests on F1‐scores across five molecular systems: tBu‐DBPO/Au(111), DBPH/Cu(110), DBPH/Cu(111), DBPH/Au(111) and BPN/Cu(111) at high and low resolution. The null hypothesis stated UniMR performance was equivalent or inferior to Zernike. Results showed a statistically significant advantage for UniMR (p=0.00095) with a large effect size (Cohen's d = 2.43), rejecting the null hypothesis. All analyses used Python scipy.stats library (v1.15.3).

### 2.4 Analysis of Performance Factors

We perform an ablation study for UniMR, as detailed in **Table** **6**. The results demonstrate that UniMR achieves the highest performance across all datasets when all modules are active. For example, on BPN‐LR, UniMR attains an accuracy of 0.91, significantly outperforming configurations without normalization (0.69) or using only original pixel features (0.75). The “Pixel‐only” approach demonstrates excellent performance in systems with significant structural differences (tBu‐DBPO and DBPH), while the “CLIP‐only” method achieves optimal results in the BPN system containing twin molecules. Additional experiments show that architectural and pre‐training choices significantly impact performance: replacing the default ViT‐B/32 encoder with ResNet‐50 (“w/RN50”) causes substantial performance degradation (0.76), whereas employing Masked Autoencoder pre‐training (“w/MAE”) yields competitive results (0.85), though still inferior to the complete UniMR framework. These comparative results robustly validate our proposed adaptive feature selection module, which intelligently selects the most suitable feature extraction strategy based on the structural characteristics of different molecular systems, thereby significantly enhancing the model's recognition performance.

**Table 6:** Ablation experiment for UniMR on four molecular systems, reported in F1‐score. “‐Norm” stands for the deactivation of the molecular normalization. “CLIP‐only” stands for semantic‐level encoding using CLIP embedding, and “Pixel‐only” stands for the raw pixel features provided by CLIP preprocessing. “w/RN50” and “w/MAE” denote replacing the default encoder with ResNet‐50 and using Masked Autoencoder pre‐training, respectively. LR: Low Resolution (10 pixels per nm), HR: High Resolution (20 pixels per nm).

	UniMR	‐Norm	CLIP‐only	Pixel‐only	w/ RN50	w/ MAE
BPN‐LR	**0.91**	0.69	0.91	0.75	0.67	0.87
BPN‐HR	**0.95**	0.71	0.95	0.70	0.70	0.87
tBu‐DBPO	**0.93**	0.73	0.73	0.93	0.73	0.73
DBPH	**0.93**	0.66	0.68	0.83	0.93	0.91
Average	**0.93**	0.70	0.82	0.80	0.76	0.85

**Figure 4:**
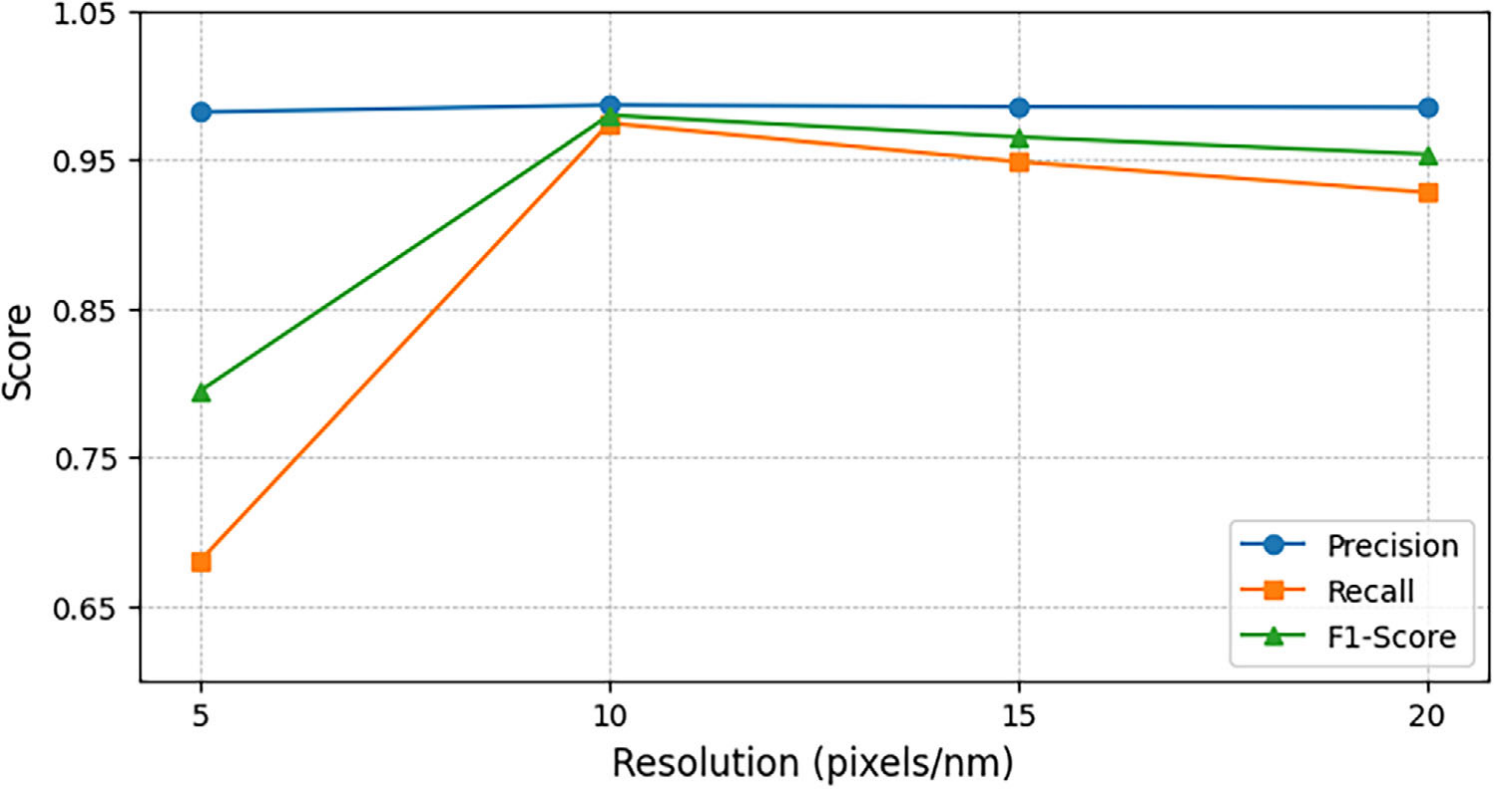
Influence of image resolution on the performance of UniMR on BPN/Cu(111). Here, images of lower resolutions are obtained by downsampling the high‐resolution (20 pixels per nm) overview STM image.

Next, we systematically evaluate the model's performance with respect to two key parameters: imaging resolution and the similarity threshold used in the matching module. As shown in **Figure** **4**, reducing the STM image resolution to one‐quarter of the original size results in only a 17% decrease in the model's F1‐score. Remarkably, even at the lowest resolution, where individual molecules are represented by merely 30 pixels, making visual distinction challenging even for human observers, our model maintains a robust F1‐score of 0.80. This demonstrates exceptional tolerance to low‐resolution imaging conditions.

**Figure 5:**
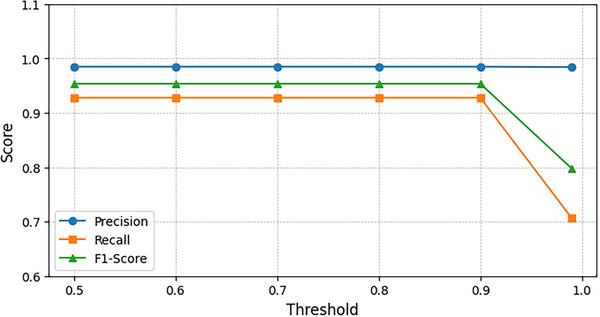
Influence of similarity threshold on the performance of UniMR on BPN/Cu(111) with high imaging resolution (20 pixels per nm).

We further investigate the impact of the similarity threshold, a critical parameter in the matching module. **Figure** **5** reveals that UniMR's recognition performance remains stable across threshold values below 0.9, with peak performance achieved precisely at this threshold. Consequently, we adopt the threshold = 0.9 as the optimal setting for all experiments.

**Figure 6:**
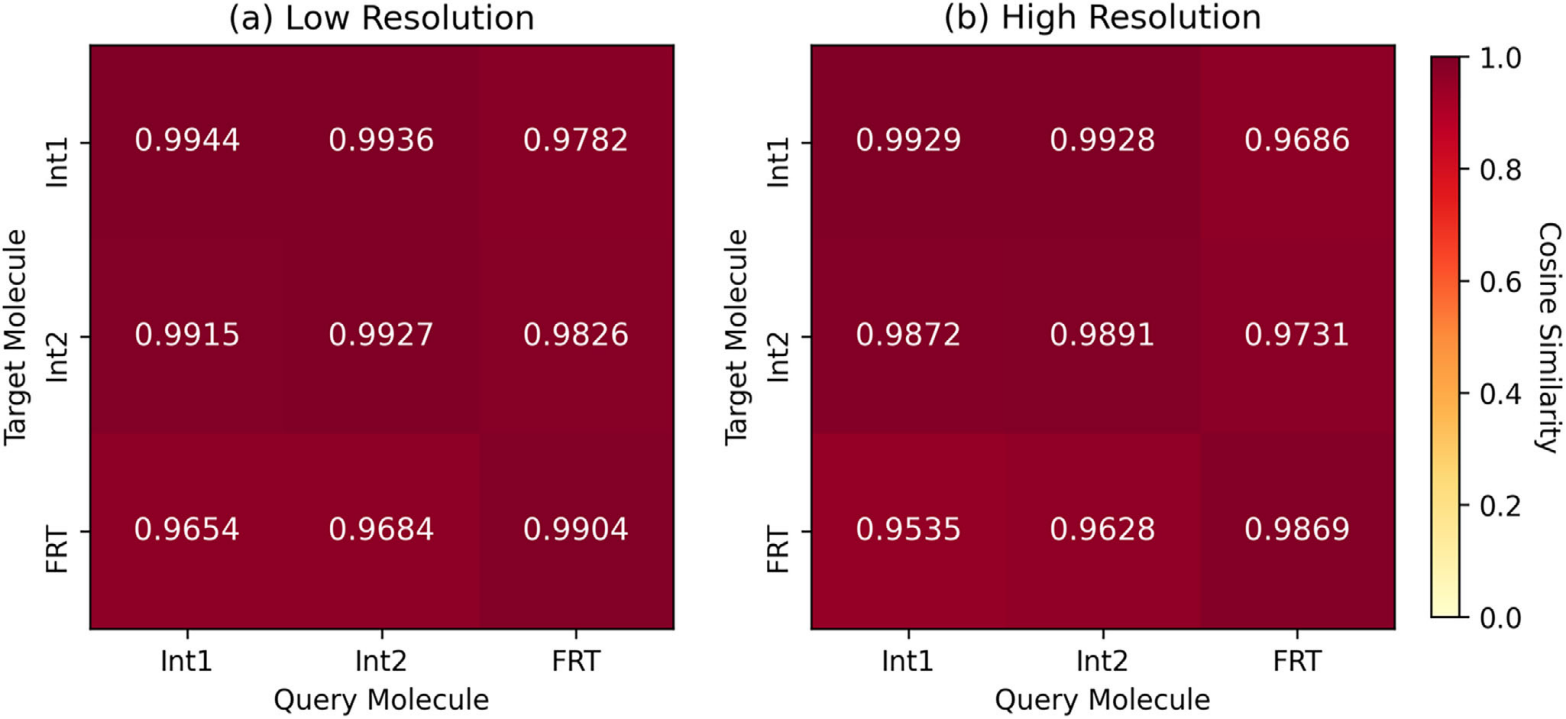
Cosine similarity analysis between target and query molecules in the BPN/Cu(111) system at low (10 pixels per nm) and high (20 pixels per nm) resolution.

**Figure 7:**
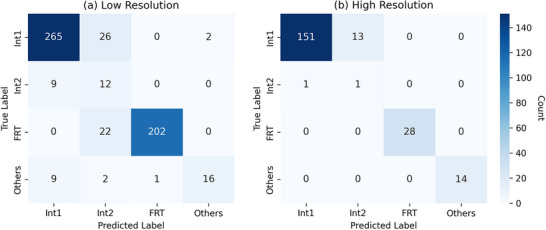
The confusion matrices on the performance of UniMR on BPN/Cu(111) with low resolution (10 pixels per nm) and high resolution (20 pixels per nm).

To further elucidate the performance disparities between molecular categories, we analyzed UniMR's recognition patterns on the BPN system using inter‐class cosine similarities and confusion matrices (**Figures** **6** and **7**). The cosine similarity, computed as the average of cosine values between the encoded target molecule and all query molecules within the same category after feature adaptive selection, reveals that both Int1 and Int2 query molecules exhibit higher similarity to target Int1 than to target Int2. This asymmetry causes Int2 molecules to be more frequently misclassified as Int1, whereas Int1 molecules are seldom misclassified as Int2, a pattern clearly reflected in the confusion matrix, where a substantial rate of Int2 samples are incorrectly assigned to Int1. As a result, Int2 achieves lower recall (0.57 in low resolution and 0.50 in high resolution) compared to Int1 (0.90 and 0.92, respectively). Additionally, the precision of Int1 is substantially higher (0.94 and 0.99) than that of Int2 (0.19 and 0.07), exacerbated by the larger population of Int1 query molecules, which skews the precision metrics. In contrast, FRT molecules, being structurally distinct from both Int1 and Int2, maintain high precision and recall across resolutions, underscoring their clear discriminability and the model's effectiveness in handling well differentiated categories.

While UniMR demonstrates robust performance in molecular recognition, its accuracy depends on the completeness of pre‐labeled reference molecules in the input image. Specifically, the model achieves optimal results usually when all molecular species present in the image are annotated in advance, that is, isolated annotation of a single target molecule is insufficient for reliable identification. This requirement stems from the framework's reliance on comparative feature analysis across different molecular structures. Nevertheless, compared to traditional approaches requiring full‐image manual annotation, UniMR significantly reduces labeling effort, as only one representative instance per molecular type needs to be marked, a tractable trade‐off for most practical applications. It is worth noting that molecular systems can exhibit further complexity, such as when a single molecular species adsorbs in multiple configurations due to different surface binding sites or energetically similar orientations, as illustrated in studies like Alldritt et al.^[^ ^54^ ^]^ In such scenarios, annotating representative instances for each distinct configuration would allow UniMR to recognize and differentiate these states, extending its applicability to diverse adsorption structures.

## 3 Conclusion

In this paper, we have developed a plug‐and‐play deep learning framework to recognize various molecules in STM images with different resolutions automatically. This framework utilizes two off‐the‐shelf models: CLIP for molecular semantic representation and GMM for feature adaptive selection. Extensive experiments on five distinct molecular systems demonstrate the method's robust performance. Given the critical role of STM in surface molecular science, our framework offers significant potential to accelerate research in supramolecular assembly, surface reaction dynamics, and low‐dimensional material characterization. Additionally, we envision its utility in streamlining STM data curation, knowledge extraction, and experimental automation.

## 4 Experimental Section

The details of the used machine learning and deep learning models are described here. The user guide of UniMR can be found in the (Figures S6–S11, Supporting Information).

### GMM

The Gaussian Mixture Model (GMM) is a probabilistic model that assumes all the data points were generated from a mixture of several Gaussian distributions with unknown parameters. GMMs are widely used in various fields, including machine learning, computer vision, and signal processing, for tasks such as clustering, density estimation, and anomaly detection.

GMM is defined as a weighted sum of K Gaussian distributions, where each Gaussian distribution is referred to as a component of the mixture. The probability density function (PDF) of a GMM is given by:

(1) $$p\left(x|\theta \right)=\sum_{k=1}^{K}\pi_{k}N\left(x|\mu_{k},\Sigma_{k}\right)$$

where x is the data point, θ represents the parameters of the GMM, which include the mixture weights $\pi_{k}$, the mean vectors $\mu_{k}$, and the covariance matrices $\Sigma_{k}$ for each component k. $\pi_{k}$ is the weight of the k‐th component, satisfying $\pi_{k}\geq 0$ and $\sum_{k=1}^{K}\pi_{k}=1$, $N(x|\mu_{k},\Sigma_{k})$ is the Gaussian distribution with mean $\mu_{k}$ and covariance matrix $\Sigma_{k}$.

In the context of the feature adaptive selection module in this paper, the GMM algorithm was employed to dynamically determine the optimal encoding method for the input data. Specifically, the GMM was used to model the distribution of the encoded data, allowing for the selection of the most informative embedding technique. By fitting the encoded data to a mixture of Gaussian distributions, the GMM provides a probabilistic framework to evaluate the distinctiveness of the encoded data. This enables the feature adaptive selection module to choose between different encoding schemes, such as using CLIP embeddings or not, based on which method yields a more pronounced separation in the GMM components. Consequently, the GMM algorithm plays a crucial role in enhancing the representational capacity and discriminative power of the encoded data, thereby improving the overall performance of the adaptive encoding module.

### ViT and CLIP

The Vision Transformer represents a paradigm shift in computer vision by adapting the transformative success of the Transformer architectures from natural language processing to image recognition tasks. Unlike traditional convolutional neural networks that rely on local receptive fields, the Vision Transformer leverages the self‐attention mechanism to process image data, enabling it to capture global dependencies within the visual input. At the heart of the ViT model is the concept of dividing an image into a set of fixed‐size patches, which are then treated as tokens for input into the Transformer architecture. This approach allows the model to process images in a manner that is inherently capable of grasping long‐range interactions and contextual information, a feature that sets it apart from CNNs, which are primarily focused on local feature extraction. One of the significant advantages of the ViT model is its adaptability and scalability, making it effective not only for image classification but also extensible to other computer vision tasks such as object detection and image segmentation. The self‐attention mechanism of ViT allows it to excel in capturing complex patterns within images by effectively managing long‐range dependencies.

Contrastive Language‐Image Pre‐training (CLIP) is an innovative model introduced by OpenAI in 2021 that builds upon such advancements in vision architectures. It represents a significant leap in multimodal learning by pre‐training on a vast dataset of image‐text pairs to learn semantic associations between images and their textual descriptions. The core concept of CLIP is to map both images and text into a shared vector space, enabling the model to understand the relationship between visual content and language. The image encoder in CLIP, which can employ either CNN‐based models like ResNet or Transformer‐based models like Vision Transformer, converts input images into feature vectors. The Vision Transformer, with its ability to process images as sequences of patches and capture global dependencies through self‐attention, serves as a powerful backbone for CLIP's image encoder. Meanwhile, the text encoder, typically implemented using a Transformer architecture, transforms textual descriptions into feature vectors. By leveraging the strengths of ViT, CLIP achieves robust performance in aligning visual and textual representations, facilitating tasks such as zero‐shot image classification and cross‐modal retrieval. The combination of ViT's global feature extraction capabilities and CLIP's contrastive learning framework underscores the potential of Transformer‐based architectures in advancing multimodal understanding.

## Conflict of Interest

The authors declare no conflict of interest.

## Supporting information



Supporting Information

## Data Availability

The data that support the findings of this study are available from the corresponding author upon reasonable request.
