# Supplementary material for: UniMR: A Plug‐and‐Play Framework of Automated Molecular Recognition for Scanning Tunneling Microscopy
Source: Adv Sci (Weinh). 2025 Dec 15;13(11):e16428. doi: 10.1002/advs.202516428 (PMC12931239; doi:10.1002/advs.202516428)
Supplement: Supplementary file 1 — Supporting Information [file ADVS-13-e16428-s001.pdf]

Supporting Information for UniMR: A Plug-and-Play Framework of Automated Molecular Recognition for Scanning Tunneling Microscopy

Ziqiang Cao,† Lingyin Zhang,† Bingzheng Wu,‡ Haonan Chen,† Junhao Sun,¶ Linghao Yan,¶ Wenfei Li,¶ Yangyang Wu,¶ Zhifang Wang,*,¶ Qigang Zhong,*,¶ and Lifeng Chi*,¶

†*School of Computer Science and Technology, Soochow University, Suzhou 215123, China*

‡*Research Center for Chemical Theory, Department of Chemistry, Fudan University, Shanghai 200438, China*

¶*State Key Laboratory of Bioinspired Interfacial Materials Science, Institute of Functional Nano & Soft Materials (FUNSOM), Soochow University, Suzhou 215123, China*

E-mail: zfwang@suda.edu.cn; qgzhong@suda.edu.cn; chilf@suda.edu.cn

Table of Contents

1. DFT Calculations and STM Simulations

2. Image Segmentation

3. Otsu Method

4. Gaussian Mixture Model (GMM)

5. Manual Molecular Recognition

6. UniMR Molecular Recognition

7. Supplementary Analysis of Performance Factors

8. User Guide of UniMR

1. DFT Calculations and STM Simulations

The electronic and geometric structures were calculated using the Vienna Ab-initio Simulation Package (VASP)[1–4] with the same parameters as in our previous studies.[5] Briefly, the Perdew-Burke-Ernzerhof exchange correlation functional,[6] augmented with Grimme's D3 dispersion correction with Becke-Johnson damping,[7,8] was employed. A plane wave basis set with an energy cutoff of 425 eV was used to expand the valence electrons, and the projector augmented-wave pseudopotentials[9,10] were adopted to describe the core electrons. For geometry optimizations, a Γ-centered 3×3×1 k-point mesh was applied. Meanwhile, for single point calculations, a denser 6×6×1 k-point mesh was used, along with a dipole correction applied along the z-direction. Based on these calculations, all STM images were simulated within the framework of the modified Bardeen theory,[11–14] assuming a p-wave tip for the CO-functionalized tip in the current experiments. All electronic states within 100 meV above the Fermi level were integrated, and the STM images were calculated by the constant-current mode, consistent with the measurement conditions. To account for finite mapping resolution in practical measurements, all calculated STM images were filtered by a Gaussian smearing with a bandwidth of 0.5 Å. An isovalue of 6×10^{-6} $e/\text{Å}^{-3}$ was adopted for the constant-current scanning mode in all simulations.

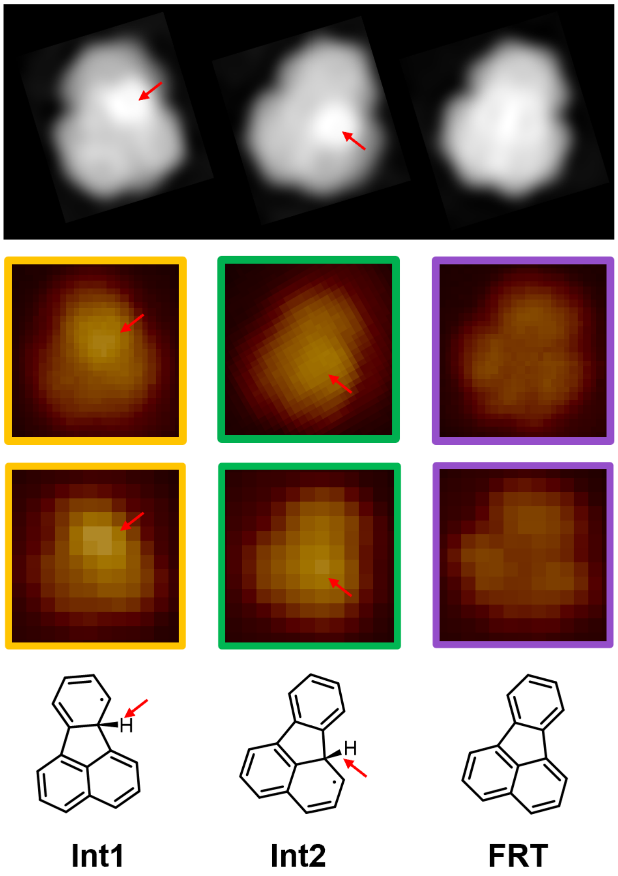

Figure S1: Simulated STM images (top row), experimental STM images (two rows in the middle with imaging resolution of 20 pixels/nm and 10 pixels/nm, respectively) and chemical structures (bottom row) of three different molecules (Int1, Int2 and FRT) with similar skeletons. Both the simulated and experimental STM images were acquired using a CO tip in constant-current mode with a sample bias of 100 mV. The experimental STM images are cut-outs from overview STM images used in this study. The protruding hydrogen atoms in Int1 and Int2 are marked with red arrows.

2. Image Segmentation

We use the findContours function to segment molecular images. The function is a pivotal component of the OpenCV library, a comprehensive computer vision and machine learning software library. This function is instrumental in detecting contours in binary images, which are defined as the boundaries of connected white or black regions within an image. It plays a crucial role in various applications such as object detection, image segmentation, and feature extraction. The findContours function operates on binary images to identify and return the contours. It accepts three parameters: the binary image, a contour retrieval mode, and a contour approximation method. The function returns two values: a list of contours and a hierarchy matrix that describes the relationship between contours. For example, the result of image segmentation for a part of an overview STM image of BPN/Cu(111) is shown in Figure S2. The underlying implementation of the findContours function is based on the Suzuki85 algorithm, which is an efficient contour tracking algorithm.

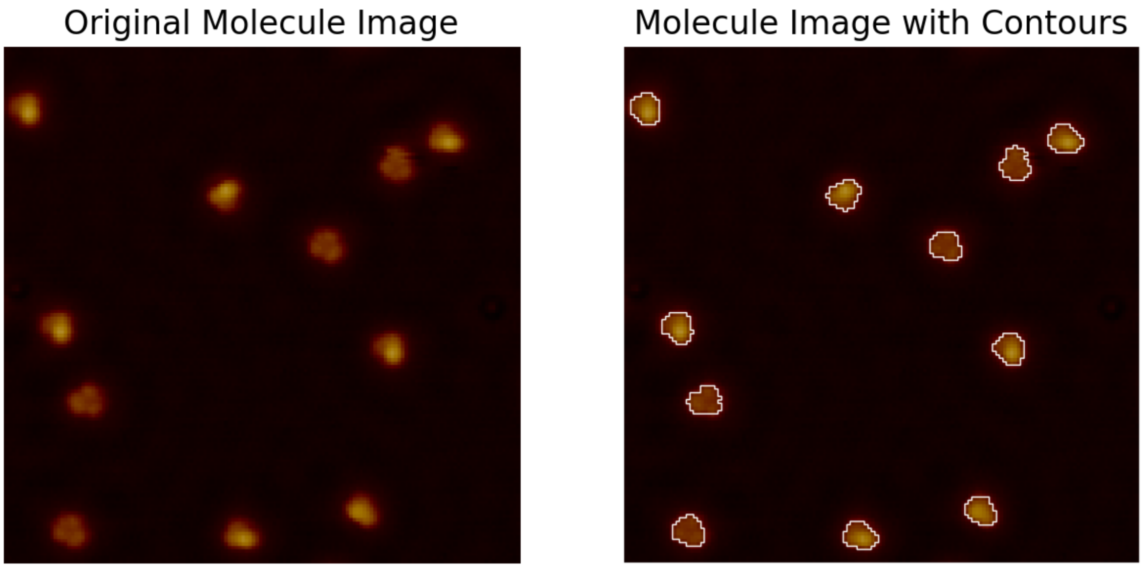

Figure S2: Molecular edge detection using the findContours function. The left panel shows an original STM image of the BPN/Cu(111) system, while the right panel presents the same image with the edges of all molecules marked by white solid lines. Based on the detected molecular edges, we cut out rectangular areas each with a single molecule in its center for subsequent processing.

3. Otsu Method

Otsu's method is an automatic threshold selection technique used for image segmentation. It determines the optimal threshold for image segmentation by maximizing the between-class variance. The method assumes that the image consists of two classes (foreground and background) and finds the optimal threshold by calculating the between-class variance for each possible threshold.

In our software, Otsu's method is used to calculate the optimal threshold for the similarity distribution of molecular images. By converting similarity values into integers ranging from 0 to 255, the software calculates the between-class variance and selects the threshold that maximizes this variance.

4. Gaussian Mixture Model (GMM)

The Gaussian Mixture Model (GMM) is a probabilistic model used to represent sample data with multiple Gaussian distributions. GMM estimates the parameters (mean, variance, and weight) of each Gaussian distribution using the Expectation-Maximization (EM) algorithm, thereby modeling the data. In our software, GMM is used to analyze the similarity distribution of molecular images. By fitting two Gaussian distributions, the software can identify the two main groups in the similarity distribution and calculate their intersection as the threshold. This method is particularly useful for complex similarity distributions, allowing for more accurate threshold determination for molecular classification.

5. Manual Molecular Recognition

In Figure 2 of the main text, we have provided some manually labeled overview STM images. Here, we provide the remaining parts that are used in this work (Figure S3).

,

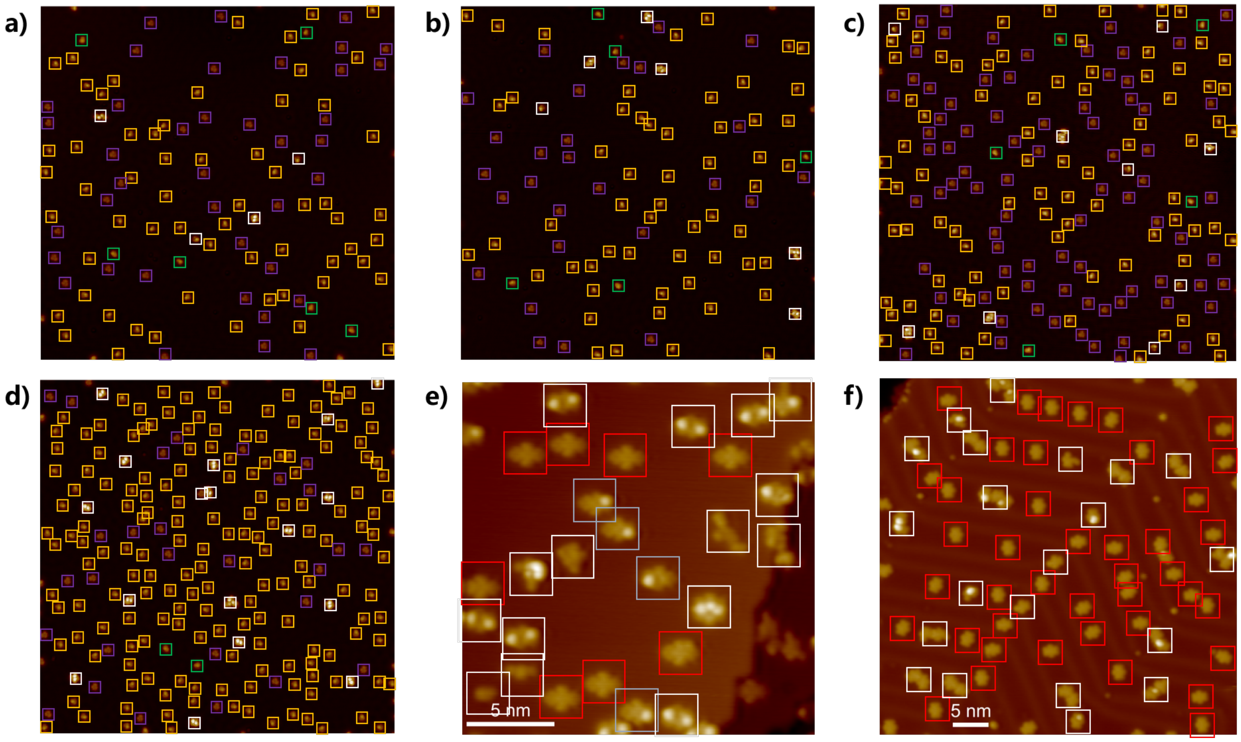

Figure S3: Overview STM images showing the results of manual molecular recognition for three molecular systems. (a–c) BPN/Cu(111) at low resolution (10 pixels/nm); (d) BPN/Cu(111) at high resolution (20 pixels/nm); (e) DBPH/Cu(110); (f) DBPH/Au(111). The colored square boxes mark the manually identified target molecules, while the white boxes mark other non-target molecules (Yellow: Int1; Green: Int2; Purple: FRT; Red: DBPH; Gray: dDBPH).

6. UniMR Molecular Recognition

In Figure 3 of the main text, we have provided the results of UniMR molecular recognition for a low-resolution overview STM image of BPN/Cu(111). Here, we provide the results for the other four STM images of BPN/Cu(111) that are tested in this work (Figure S4). Additionally, we provide UniMR recognition results for the other four molecular systems investigated: DBPH/Cu(110), DBPH/Au(111), tBu-DBPO/Au(111), and DBPH/Cu(111), as shown in Figure S5. The precision, recall, and F1-score of UniMR molecular recognition can be determined by comparing with the results of manual molecular recognition (Figure S4 vs Figure S3 (a-d); Figure S5 vs Figure S3 (e-f) and Figure 2 (a-b)).

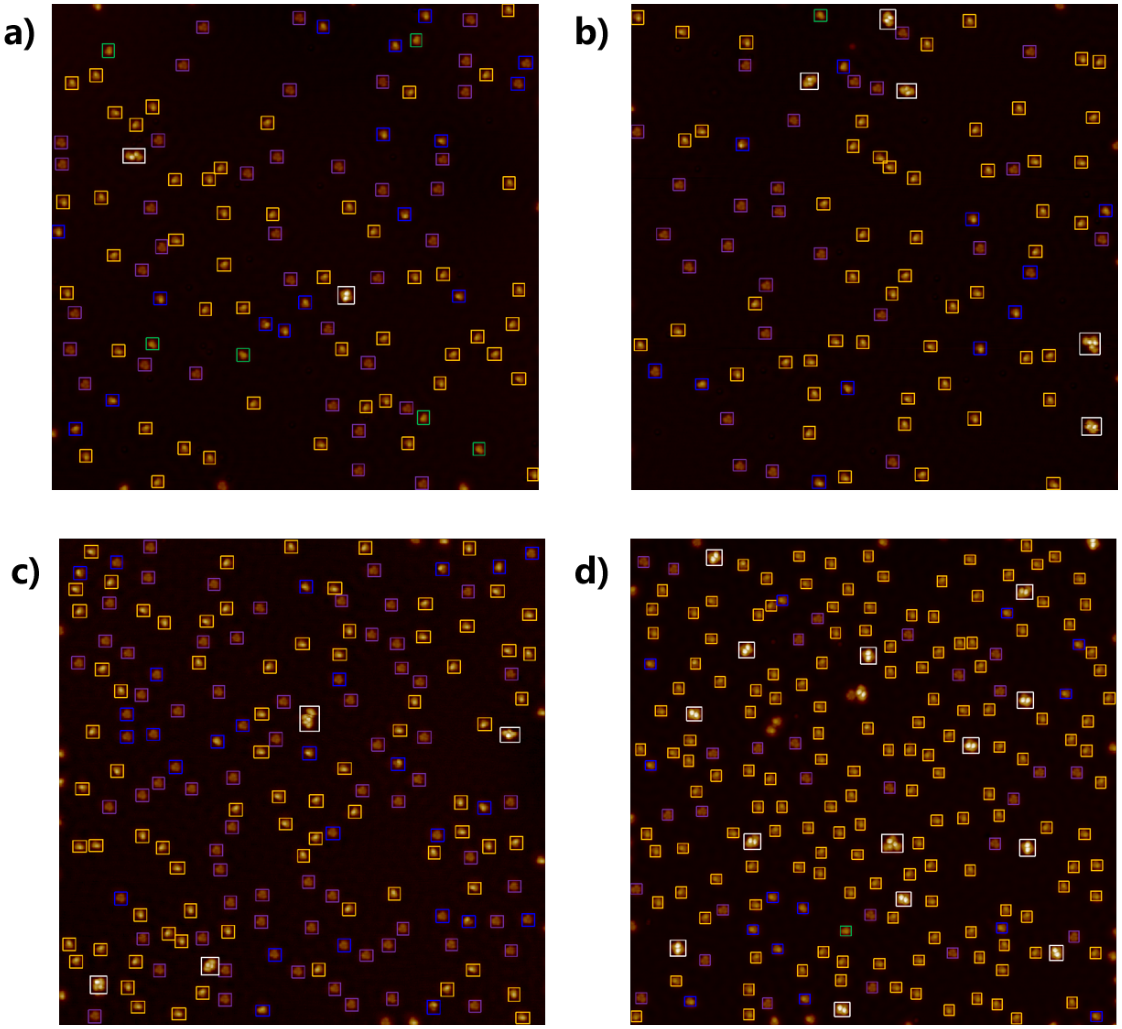

Figure S4: The UniMR molecular recognition results for STM images of BPN/Cu(111). The blue square boxes represent incorrect matches, while the other colors represent correct matches (Yellow: Int1; Green: Int2; Purple: FRT; White: Others). The precision, recall, and f1-score for the four images are listed as follows. (a) Precision: 0.95, recall: 0.87, F1-score: 0.89. (b) Precision: 0.90, recall: 0.89, F1-score: 0.89. (c) Precision: 0.91, recall: 0.86, F1-score: 0.88. (d) Precision: 0.98, recall: 0.93, F1-score: 0.95.

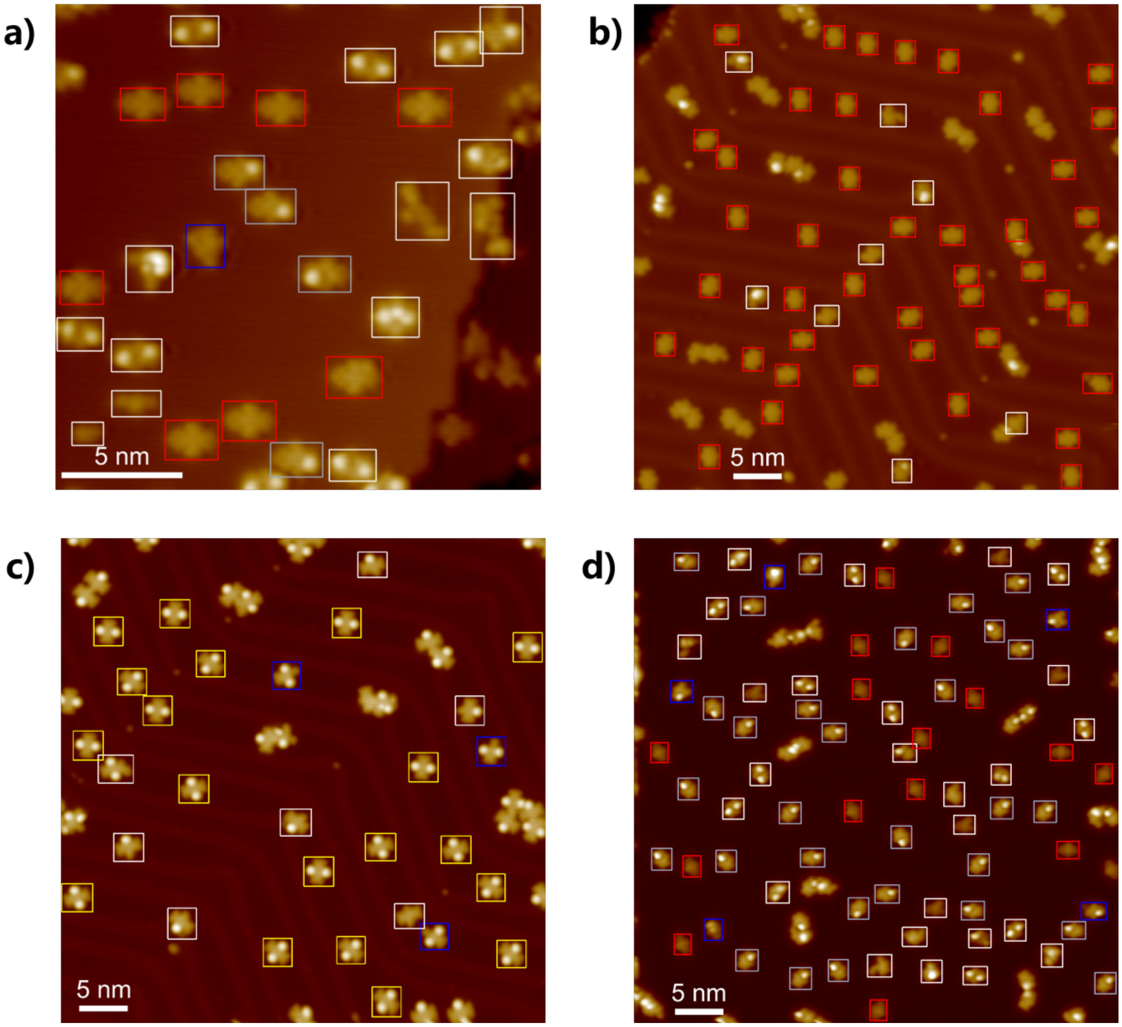

Figure S5: The UniMR molecular recognition results for STM images of (a) DBPH/Cu(110), (b) DBPH/Au(111), (c) tBu-DBPO/Au(111), (d) DBPH/Cu(111). The blue square boxes represent incorrect matches, while the other colors represent correct matches (Red: DBPH; Gray: dDBPH; Yellow: tBu-DBPO; White: Others). The precision, recall, and f1-score for the four images are listed as follows. (a) Precision: 0.92, recall: 1.00, F1-score: 0.96. (b) Precision: 0.84, recall: 1.00, F1-score: 0.91. (c) Precision: 0.86, recall: 1.00, F1-score: 0.93. (d) Precision: 0.90, recall: 0.98, F1-score: 0.94.

7. Supplementary Analysis of Performance Factors

The performance metrics in Table S1 indicate that molecular coverage has no consistent or significant impact on UniMR's overall performance. The weighted average F1-scores remain stable at 0.89 (0.07 ML), 0.88 (0.11 ML), and 0.94 (0.13 ML) across different coverage levels. Although minor fluctuations occur for specific molecular types like Int1, Int2, and FRT, these variations show no clear correlation with increasing coverage. This demonstrates the robustness of UniMR across varying molecular coverage and its applicability in diverse experimental scenarios.

Table S1: Influence of molecular coverage on the performance of UniMR on BPN/Cu(111) with low imaging resolution (10 pixels/nm).

Molecular coverage		Precision	Recall	F1-score
0.07ML	Int1	0.91	0.93	0.92
	Int2	0.17	0.20	0.18
	FRT	1.00	0.93	0.96
	Average	0.90	0.89	0.89
0.11ML	Int1	0.88	0.99	0.93
	Int2	0.00	0.00	0.00
	FRT	1.00	0.80	0.89
	Average	0.91	0.86	0.88
0.13ML	Int1	0.98	0.89	0.93
	Int2	0.26	1.00	0.42
	FRT	0.99	0.97	0.98
	Average	0.96	0.93	0.94

8. User Guide of UniMR

This software is designed to segment, enhance, and match molecular images using image processing and machine learning techniques. Users can upload molecular images, select target molecules, and obtain classification results.

Recommended Environment For optimal compatibility, we recommend installing the following dependencies with our verified versions:

Python: 3.9.0

CUDA Toolkit: 12.4

PyTorch: 2.5.1 (with CUDA 12.4 support)

Here are the official installation guidelines for CUDA and PyTorch:

CUDA: https://docs.nvidia.com/cuda/cuda-installation-guide-microsoft-windows/index.html

PyTorch: https://pytorch.org/get-started/locally/

Here are the download links for the corresponding versions:

Python 3.9.0: https://www.python.org/downloads/release/python-390/

CUDA Toolkit: https://docs.nvidia.com/cuda/cuda-installation-guide-microsoft-windows/index.html

PyTorch 2.5.1 (CUDA 12.4): https://pytorch.org/get-started/previous-versions/#v251

Dependencies Please download the compressed package from https://github.com/LingyinZhang/UniM[illegible] and unzip it to your desired installation location. Then open Windows PowerShell in that folder.

We recommend creating a virtual environment using conda. For conda installation, please refer to the following documentation:

https://docs.conda.io/projects/conda/en/latest/user-guide/install/index.html

Requirements

matplotlib==3.9.2

numpy==1.26.3

openai-clip==1.0.1

opencv-python==4.10.0.84

pillow==10.2.0

scipy==1.13.1

torch==2.7.1

torchvision==0.22.1

scikit-learn==1.6.1

To install the required dependencies, execute this command:

pip install -r requirements.txt

Usage In this section, we will introduce how to use the UniMR software.

1. Launch the UniMR Software. Upon running the software, the Start Window will appear(Figure S6). Ensure that your Python environment is properly configured and that all dependent libraries are installed.

2. Load Molecular Images. In the Start Window, click the "Select Image Path" button to select the file path containing the target molecular image, and then clik "Confirm" button. The software will load the image and display it in the Segmentation Window(Figure S7).

3. Resize the segmentation. In the Segmentation Window, you can adjust the segmentation effect through the Threshold, Min Area, and Max Area. After adjusting, click the "Apply Settings" button to enter the Labeling and Classification Window(Figure S8).

4. Label Target Molecules. In the Labeling and Classification Window, click the "Select Target Molecule" button, then click on the molecule you want to use as a sample in the image. The software will prompt you to enter the target molecule's name and color(Figure S9). Red, Green and Blue respectively represent the three primary colors of RGB. Please enter an integer between 0 and 255. After entering the details, click the "Submit" button, then the target molecule will be marked and saved. Multiple targets can be selected one by one. The options on the right default to the Settings applied in the paper. You can also adjust

them by yourself.

5. Classify Molecules. Click the "Start Classification" button to start classifying, and the software will vectorize all molecules and match them with the target molecules. If the match result is above the set threshold, the molecule will be classified as the corresponding target molecule; otherwise, it will be classified as "other".

6. View Results. Please wait patiently. When the classification is completed, the software will pop up a Classification Results Window showing the quantity of each type of molecule(Figure S10). Meanwhile, the classification results will be synchronously updated to the image in the Labeling and Classification Window(Figure S11).

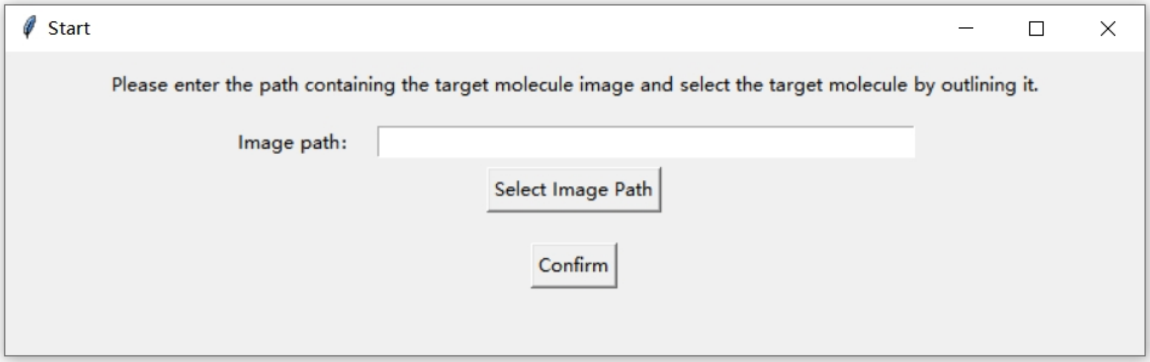

Figure S6: The Start Window. It is the initial interface of the software, including image path input fields and buttons.

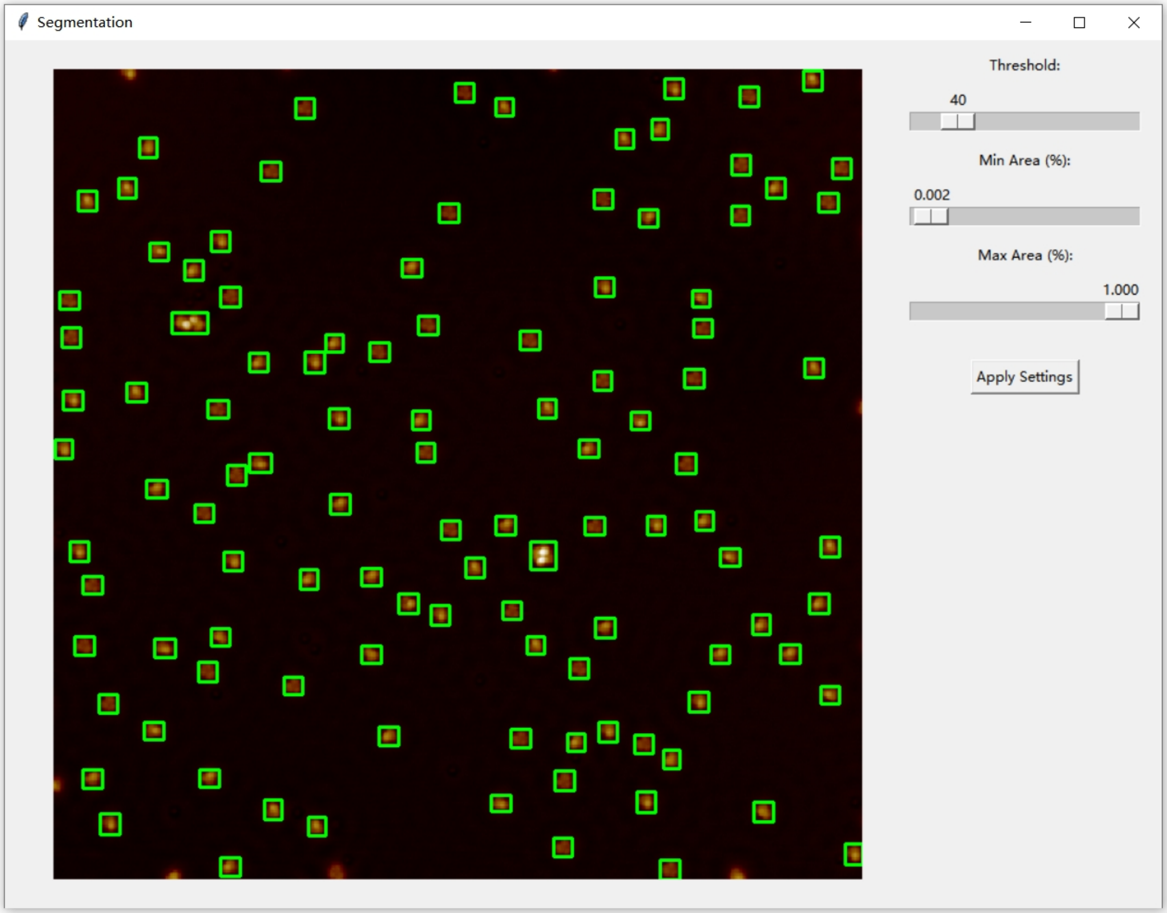

Figure S7: The segmentation Window. This window helps optimize image segmentation for accurate molecular recognition.

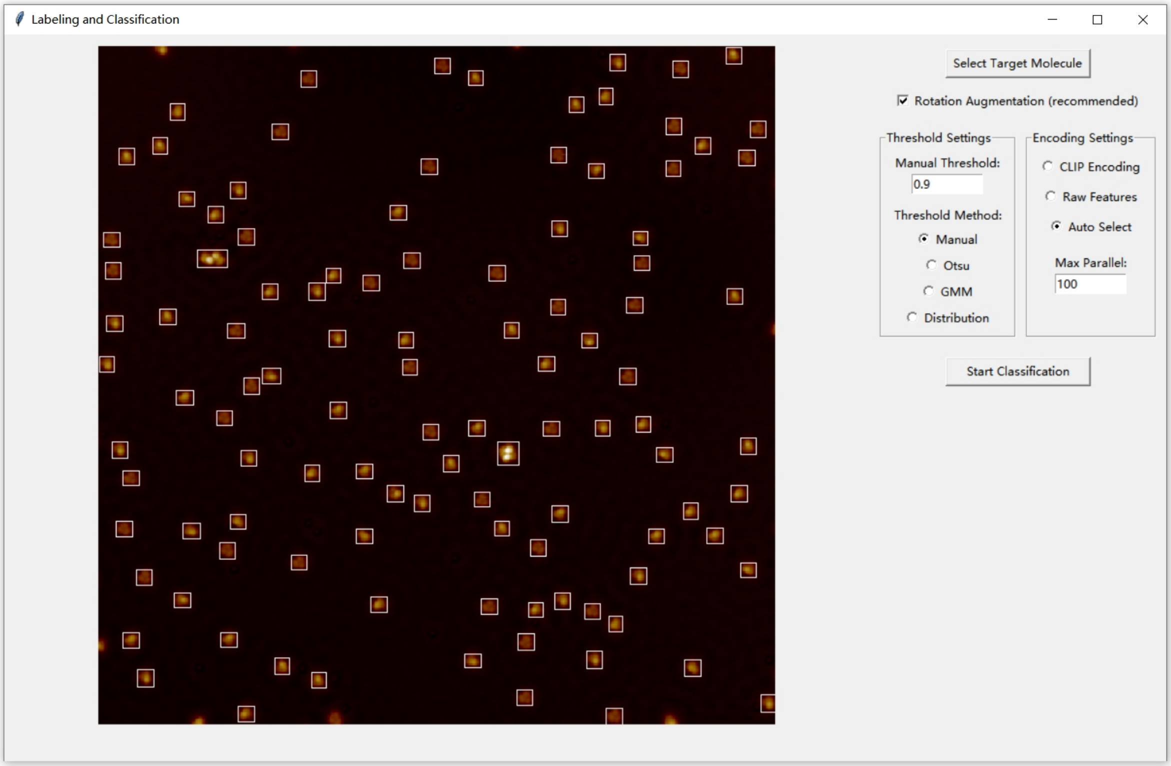

Figure S8: Labeling and Classification Window. This window is essential for visualizing and managing molecular recognition results.

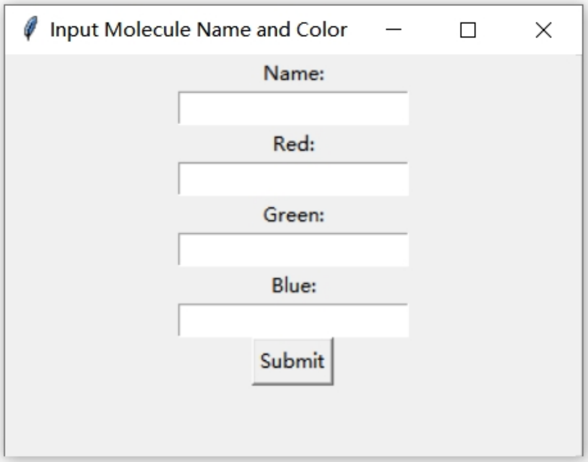

Figure S9: Target Select Window. This window allows you to name the target molecule you just clicked on and select the color for marking.

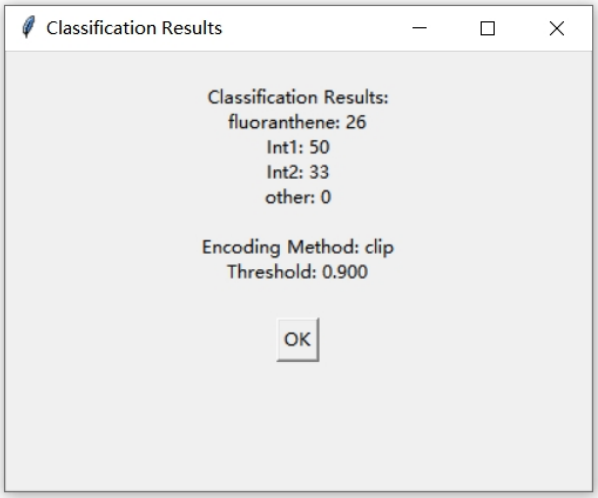

Figure S10: Classification Results Window. This window displays classification results, including the count of each type of molecule.

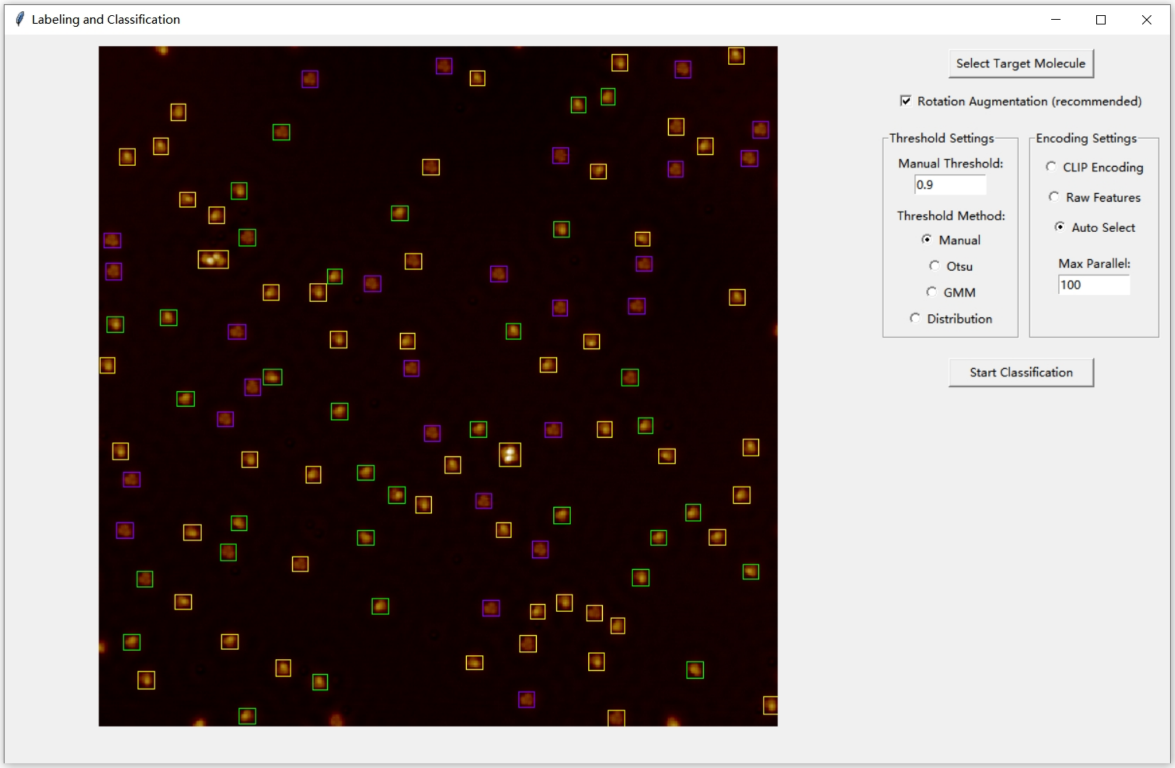

Figure S11: Image Classification Results. This window displays the image of the classification results, marked with the color previously indicated by the user.

References

(1) Kresse, G.; Hafner, J. Ab initio molecular dynamics for liquid metals. *Physical review B* **1993**, *47*, 558.

(2) Kresse, G.; Furthmüller, J. Efficient iterative schemes for ab initio total-energy calculations using a plane-wave basis set. *Physical review B* **1996**, *54*, 11169.

(3) Kresse, G.; Hafner, J. Ab initio molecular-dynamics simulation of the liquid-metal–amorphous-semiconductor transition in germanium. *Physical Review B* **1994**, *49*, 14251.

(4) Kresse, G.; Furthmüller, J. Efficiency of ab-initio total energy calculations for metals and semiconductors using a plane-wave basis set. *Computational materials science* **1996**, *6*, 15–50.

(5) Zhong, Q.; Jung, J.; Kohrs, D.; Kaczmarek, L. A.; Ebeling, D.; Mollenhauer, D.; Wegner, H. A.; Schirmeisen, A. Deciphering the mechanism of on-surface dehydrogenative C–C coupling reactions. *Journal of the American Chemical Society* **2024**, *146*, 1849–1859.

(6) Perdew, J. P.; Burke, K.; Ernzerhof, M. Generalized gradient approximation made simple. *Physical review letters* **1996**, *77*, 3865.

(7) Grimme, S.; Antony, J.; Ehrlich, S.; Krieg, H. A consistent and accurate ab initio parametrization of density functional dispersion correction (DFT-D) for the 94 elements H-Pu. *The Journal of chemical physics* **2010**, *132*, 154104.

(8) Grimme, S.; Ehrlich, S.; Goerigk, L. Effect of the damping function in dispersion corrected density functional theory. *Journal of computational chemistry* **2011**, *32*, 1456–1465.

(9) Blöchl, P. E. Projector augmented-wave method. *Physical review B* **1994**, *50*, 17953.

(10) Kresse, G.; Joubert, D. From ultrasoft pseudopotentials to the projector augmented-wave method. *Physical review B* **1999**, *59*, 1758.

(11) Bardeen, J. Tunnelling from a many-particle point of view. *Physical review letters* **1961**, *6*, 57.

(12) Duan, S.; Zhang, I. Y.; Xie, Z.; Xu, X. Identification of water hexamer on Cu (111) surfaces. *Journal of the American Chemical Society* **2020**, *142*, 6902–6906.

(13) Duan, S.; Tian, G.; Xu, X. A general framework of scanning tunneling microscopy based on Bardeen's approximation for isolated molecules. *JACS Au* **2022**, *3*, 86–92.

(14) Zhu, Y.; Xue, R.; Ren, H.; Chen, Y.; Yan, W.; Wu, B.; Duan, S.; Zhang, H.; Chi, L.; Xu, X. Reconstructing Pristine Molecular Orbitals from Scanning Tunneling Microscope Images via Artificial Intelligence Approaches. *JACS Au* **2025**, *5*, 3163–3170.
